# Exertional heat illness: international military-oriented lessons learned and best practices for prevention and management

**DOI:** 10.3389/fphys.2025.1456984

**Published:** 2025-03-05

**Authors:** Yoram Epstein, Nisha Charkoudian, David W. DeGroot, Carol House, Itay Ketko, Lydia Yu Li Law, Alexandra Malgoyre, Francis O’Connor, Omar Tayari, Jason Kai Wei Lee

**Affiliations:** ^1^ School of Public Health, Faculty of Medicine, Tel Aviv University, Tel Aviv, Israel; ^2^ Israel Defense Forces Medical Corps, Surgeon General’s Headquarters, Ramat Gan, Israel; ^3^ Thermal and Mountain Medicine Department, US Army Research Institute of Environmental Medicine, Natick, MA, United States; ^4^ US Army Heat Center, Ft Moore, GA, United States; ^5^ UK Armed Forces Heat Illness Clinic, Portsmouth, United Kingdom; ^6^ Department of Military Medicine, Faculty of Medicine, Hebrew University, Jerusalem, Israel; ^7^ Heat Resilience and Performance Centre, Yong Loo Lin School of Medicine, National University of Singapore, Singapore, Singapore; ^8^ Operational Environment Department, French Armed Forces Biomedical Research Institute/IRBA, Brétigny surOrge, France; ^9^ Exercise Biology for Performance and Health Laboratory (LBEPS), University Evry-Paris Saclay, Evry, France; ^10^ Ecole du Val de Grace, French Military Medical Academy, Paris, France; ^11^ School of Medicine, Uniformed Services University of Health Sciences, Bethesda, DC, United States; ^12^ Human Potential Translational Research Programme, Yong Loo Lin School of Medicine, National University of Singapore, Singapore, Singapore; ^13^ Department of Physiology, Yong Loo Lin School of Medicine, National University of Singapore, Singapore, Singapore

**Keywords:** climate change, exertion, heat tolerance, exertional heat stroke, military, return to duty

## Abstract

Climate change has resulted in more frequent and intense heat waves, leading to elevated global temperatures and posing a significant health threat to individuals working in hot environments such as military personnel. Ensuring both safety and performance, alongside the increasing risk of exertional heat illnesses (EHI) due to rising temperatures, is hence even more crucial. Extensive research conducted over many years has aimed to understand the causes and impacts of EHI and develop prevention and treatment strategies. This review summarizes the research on the impacts of heat on health and performance in military settings, consolidates evidence-based strategies for EHI prevention and pre-hospital management, summarizes sex differences in heat tolerance, and discusses best practices for recovery and return to duty post-EHI. The aim is to share the knowledge and practices derived from military research to protect the health and performance of individuals in various populations exposed to heat.

## 1 Introduction

The escalating impacts of climate change have manifested in an increased frequency and intensity of heat waves and elevated global temperatures. Environmental heat has thus gained recognition as a significant and enduring ‘silent’ threat to individuals operating in hot environmental settings including military personnel, emergency responders and outdoor workers. In military settings, for example, the United States Army reported a 42% increase in the rate of heat exhaustion cases in the last 3 years (Maule et al., 2024). The challenges of environmental heat are not new. Extensive research over decades has been conducted to understand the causes and impacts, and strategies for prevention and treatment of exertional heat illnesses (EHI). Our goals in the present brief review are 4-fold: First, to highlight relevant research related to the impact of heat on health and performance of military personnel; second, to consolidate evidence-based strategies for prevention and pre-hospital management of EHI; third, to summarize evidence of sex differences in tolerance to environmental heat; and fourth, to discuss best practices for management of recovery and return to duty post-EHI. For more in-depth review into the specifics of heat stress physiology, performance implications, and heat illness pathophysiology, readers are directed to recent reviews by Periard et al. (2021), Cramer et al. (2022), and Bouchama et al. (2022).

## 2 Exertional heat illness

EHI remains a longstanding and significant concern, especially in physically demanding activities and hot environments. Despite the historical understanding of heat illness, the related concepts and spectrum of classifications in current definitions can be confusing due to complexities resulting from overlapping features leading to potential misdiagnosis and or delayed treatment. Accurate EHI definitions, based on research and evidence, are critical for improving clinical recognition, diagnosis, timely intervention, and management. Such revisions carry profound implications for public health, in addition to military settings.

The conventional understanding of “heat illness” is still linked to the development of an illness during exposure to a hot environment while EHI relates to strenuous exercise and elevated body temperatures. It is important to note that since strenuous exercise is associated with high metabolic heat production that cannot readily by dissipated, elevated body temperatures and EHI can still occur even in temperate environments, although environmental heat stress is still considered an adjuvant risk factor (Epstein and Yanovich, 2019; Bouchama et al., 2022).

EHI encompasses a range of disorders, varying in severity and categories of physiological dysfunction, and is often referred to as a continuum, where excessive heat strain can lead to exhaustion, injury, and heatstroke (Leon and Bouchama, 2015). The separation across conditions is not always clear, and the progression from exhaustion in the heat to heatstroke is not linear. Exertional heat stroke (EHS) is clinically characterized by central nervous system dysfunction, and often but not always extreme hyperthermia (body core temperature >40°C) (Bouchama et al., 2022; Roberts et al., 2023; Epstein and Yanovich, 2019). But for the other EHIs, there is a lack of specific temperature thresholds, symptoms, and categorization, due to limited understanding of the exact pathophysiology behind these conditions and overlapping features (Noakes, 2008).

Related to EHI is a condition termed Exercise-associated collapse (EAC). This is a transient orthostatic hypotension and is defined as the inability to stand or walk unaided during or after the completion of strenuous exercise and can occur without hyperthermia (Irelan and Schroeder, 2023). Exercise-associated collapse is generally a benign condition and additional diagnostic tests to exclude other medical conditions (e.g., cardiovascular) should be administered to confirm the diagnosis (Asplund et al., 2011).

A framework that can distinguish across conditions where there is no clear evidence of pathological changes (i.e., pathological rise in body core temperature, altered cerebral function and or other abnormal pathological changes) is therefore required to enable more appropriate diagnoses of ‘heat illnesses’, distinguishing them from those where body core temperature is abnormally elevated (Noakes, 2008; Laitano et al., 2019). The utility of biomechanical and biological markers should be explored to better differentiate between types of conditions (Geng et al., 2015; Lee et al., 2023; Tang et al., 2024). This will ensure that rapid and appropriate treatment is applied during an emergency. It should be noted that exploring biochemical pathways as biomarkers can deepen our understanding in some cases of heat tolerance mechanisms in the context of EHI. While several such biomarkers (e.g., HMOX1, ACSL4 pathways) are under investigation, it is still at a basic science level (Li et al., 2024).

## 3 Prevention of EHI

**FIGURE 1:**
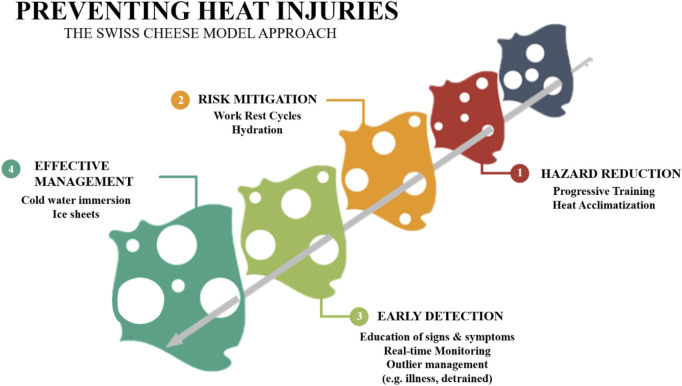
Framework to prevent heat injuries using the Swiss Cheese Model.

In order to develop a comprehensive understanding towards the prevention of heat injuries, one approach would be to adopt the established risk management model called the Swiss Cheese method to identify and analyse key potential layers of vulnerabilities. As depicted in Figure 1, heat injury prevention is made of multiple layers of effort encompassing hazard reduction, risk mitigation, early detection and effective management. In sum, hazard reduction focuses on elements such as progressive training and heat acclimatization to prepare individuals for high heat stress environments. Risk mitigation includes implementing work-rest cycles and ensuring proper hydration to minimize heat stress. Additional preventive strategies like arm immersion cooling, ice slurry ingestion and microclimate cooling (DeGroot et al., 2013; Lee et al., 2015) to alleviate heat strain can also be considered to reduce and/or attenuate heat strain. Arm immersion cooling is employed at training bases in the US Army that offers the double benefit of decreasing core temperature due to decreased metabolic rate and increased thermal comfort (DeGroot et al., 2015). Ice slurry ingestion has been adopted by the Singapore Army as a pre-activity cooling strategy for selected activities. Early detection emphasizes the importance of the education of signs and symptoms of heat-related illnesses, leveraging on technology to incorporate real-time monitoring and managing outliers to identify potential risks promptly. For example, during events that might elicit excessive hyperthermia, such as rucksack marches, long hikes, long distance running, or other endurance events, any individual exhibiting signs of extreme fatigue relative to his/her peers participating in the same activity, including not keeping up with the pace, feeling sick, displaying confusion, and unexplained muscle pain should have his/her activity stopped. This individual should be managed as a suspected case of EHI before other diagnoses are considered. Effective management involves utilizing techniques such as cold water immersion and ice sheets to treat and alleviate heat injuries efficiently. There is hence potential for such strategies to be tailored for other populations and applications.

The fundamental components of these preventive strategies should be targeted at personal and organizational levels, in addition to the mainstay of education (Epstein et al., 1999; Eifling et al., 2024; Tustin et al., 2021; Alele et al., 2020a). This provision of education and training to leaders at all levers should be an on-going mission, with emphasis on best practices for heat stress mitigation. It is important to note that a 2-to-3-year “tragedy loop” was recognized, where following a pronounced case of EHS, interest is renewed in prevention through education and training, as well as in medical management (Galer, 2019). However, with time, there is a loss of institutional memory as experienced leaders and trainers are reassigned and replaced by less experienced personnel, possibly culminating in another death.

## 4 Prehospital management of EHI

Timely identification and response to EHS is crucial for favorable outcomes. Initial triage and treatment should occur within the “golden half-hour,” prioritizing the principle of “cool first, transport second” (Heled et al., 2004). Whole-body cold-water immersion is the current gold standard treatment, as it rapidly reduces temperature, improving prognosis and lifesaving (Belval et al., 2018). Under field conditions, an alternative cooling approach, with similar cooling efficiency, is the use of a provisional cooling bag, in which less water and ice are needed (Hosokawa et al., 2017).

Where a tub of ice water is not readily available, such as at ultramarathon events or field training, iced sheets (bedsheets immersed in ice water) have yielded ‘acceptable’ cooling rates of 0.16 °C/min (DeGroot et al., 2023). These ice sheets are replaced every 3 min to maintain effectiveness (Caldwell et al., 2022). In the absence of specialized equipment, pouring large volumes (>40 L) of tap water (Hadad et al, 2004) or using commercial cooling pads placed on the torso, abdomen, arms and legs can serve as alternative practical treatments where cold-water immersion is not feasible (Tan et al., 2017).

## 5 Sex differences in the tolerance to heat

There are increasing numbers of women in the military and who participate in work or other activities involving prolonged exposure to environmental heat (Giersch et al., 2022). As such, many in the physiology community have been working towards a more complete understanding of the physiology (and potential pathophysiology) of exercise-heat stress in women. Women appear to have both advantages and disadvantages in the regulation of body temperature in the heat (Charkoudian and Stachenfeld, 2014). A potential “benefit” is a woman’s (on average) smaller body size, and therefore larger body surface area-to-mass ratio compared to men. During military or industrial activities (most of which involve weight-bearing exercise), heat is generated based on body mass (muscle mass) and dissipated based on surface area. In these circumstances, a larger ratio will be beneficial for heat dissipation in most environmental conditions (Akavian et al., 2025; Taylor et al., 2024). Importantly, this is not the case in so-called “uncompensable” environments, where environmental temperature is higher than body temperature and humidity is so high that sweat cannot evaporate from the skin. In such conditions, or when encapsulated (i.e., with protective clothing), there is no advantage to having a high body surface area-to-mass ratio (Cheung et al., 2000).

Relative advantages and disadvantages between men and women may cancel each other out when it comes to relative risk for EHS, as shown in a recent retrospective analysis of ∼5,000 active-duty U.S. soldiers where cases were compared with controls executing the same tasks in the same environments (Giersch et al., 2023). This finding aligns with prior retrospective epidemiological studies, with similar reported incidence rates for both sexes (Williams and Oh, 2022).

The broader question of sex differences in the tolerance to heat remains unresolved. Incidences of EHS increased for both sexes in the US Armed Forces from 2015 to 2018, but the incidences of other heat illnesses (e.g., heat exhaustion) were higher in women (1.30–2.89 vs. 0.98 to 1.98 per 1000-person-years in women and in men, respectively) (Alele et al., 2020b). While similar observations have been reported in some studies, there are other studies reporting higher incidences and proportions of heat illnesses in men (Gifford et al., 2019). Such variations highlight the multifactorial nature of heat illness risk, necessitating further investigation and understanding of sex differences for targeted mitigation and management strategies to be developed (Alele et al., 2020b).

## 6 Return to duty (RTD)

The decision to return an individual to duty following recovery from an EHI is complex and challenging. While it is primarily a medical decision, it often requires input from other professionals such as commanders and trainers (O’Connor et al., 2018).

In the Israel Defence Force (IDF), a thorough analysis of the medical, environmental, and organizational factors of the EHI case is conducted, with recovering soldiers undertaking further assessment and heat tolerance testing. The militaries of the UK, Singapore, and France also review EHI cases, with specific criteria guiding return-to-duty (RTD) decisions. In the UK, all cases of moderate, severe and recurrent EHI are reviewed by the Heat Illness clinic. A similar approach is taken in Singapore, with EHI cases reviewed by the Army Medical Services. In France, all EHS (but not other EHI) are registered and investigated to explore background risk factors. The unit physician can decide on RTD when the background for injury is clear. At least one extrinsic and one intrinsic risk factor must be present to consider the case as incidental and clear the soldier to RTD. All other cases are discussed with the French Armed Forces Biomedical Research Institute for further assessment and guidance in relation to RTD. Severe cases of EHS, recurrent episodes of EHI, or cases with no definitive cause are entitled for a heat tolerance test (HTT). The U.S. Army adopts a comprehensive approach, with a system that progressively reintroduces environmental and exertional acclimatization (Technical Bulletin, 2022). Initial and serial follow-up visits are conducted with medical providers, including the Army Heat Illness Clinic, with referrals for more complex cases (e.g., recurrent and failure to progress) to the Multi-disciplinary Case Review Committee at the Uniformed Services University for additional guidance. The US Military recently published a set of Clinical Practice Guidelines that includes service specifc guidance and integrates the RTD process (O’Connor et al., 2024).

Heat tolerance testing is a ‘functional test’ used as a part of the recommendation for RTD (O’Connor et al., 2010), as it assesses the individual’s thermoregulatory capacity under heat stress. The first HTT was conducted by Dreosti in the early 1930s where miners with a rapid rise in body core temperature and lower cooling rates were identified as less heat-tolerant (Dreosti, 1935). Modification of the HTT included cardiovascular and sweat rate measurements as additional indicators of strain, alongside body temperature (Wyndham et al., 1954).

Brown and colleagues explored assessing heat tolerance based on orthostatic tolerance, as a significant decrease seemed linked to thermal stress symptoms (Brown et al., 1959). This was also observed by Shvartz et al. (1977) who found superior orthostatic responses in physically fit and heat-acclimated individuals. However, this approach of gauging cardiovascular adaptability to exercise-heat exposure was deemed limited and hence abandoned. A military oriented HTT was subsequently introduced by the IDF, incorporating elements from earlier tests with subsequent refinements in the late 1980s, replacing bench stepping with treadmill walking and reducing the test duration.

To date, the IDF’s HTT (Schermann et al., 2018) and UK’s Heat Tolerance Assessment (HTA) (House et al., 2023) are the two protocols that have been used or adapted to provide insights to thermoregulatory function in the militaries of France, Singapore, and the United States of America (Lee et al., 2017; Jones and Heaney, 2017). Through the IDF-HTT and HTA, the IDF and UK return on average 90% and 78% of individuals respectively to full operational duties. Individuals that fail the first HTT typically undergo repeat assessment (up to three) before they are either medically discharged or referred for further medical investigations (Gardner et al., 2020). Experience from such heat tolerance testing highlight that (a) most EHI cases result in a temporary condition of heat intolerance; (b) intersubject variability exists in recovery time of the thermoregulatory system; and (c) high sensitivity of the IDF-HTT. It should be noted that the UK-HTA is a more military-task oriented test, since the test includes carrying weight and the subject is dressed in military combat clothing. The IDF-HTT is conducted with young male soldiers with an average-high fitness level. The results might have a selection bias and must hence be re-evaluated for other populations. Both tests do not account for individual variability in body composition or heat acclimatization, although the UK-HTA does account for physical fitness. In this context, U.S. investigators have recently shown that heat acclimation *per se* improves heat tolerance using the IDF-HTT protocol, which may provide more insight into the physiological mechanisms involved (Mitchell et al., 2021).

There is interest in industrial communities in the development of occupational HTTs, particularly for individuals working in progressively hotter climates and individuals working in uncompensable environments (e.g., those in encapsulated clothing). For example, a test for nuclear power stations workers was developed (Kenney et al., 1986) measuring heart rate recovery 5 minutes after exercise cessation. The authors concluded that for the most intense work-heat conditions encountered in nuclear power stations, the physiological limit (in minutes) could be predicted with 95% confidence. A similar attempt was made to assess the validity and reliability of a varying workload test for fire fighters (Watkins et al., 2018). In terms of enhancing assessments, French investigators are studying sweat efficiency as an additional insight to understand origin of heat intolerance and the reversibility of intolerance. It was demonstrated that the HTT had a 100% negative predictive values for future EHI over 2 years of follow-up for Servicemembers with a history of recurrent heat injury and negative HTT results (Kester et al., 2024).

With growing evidence that heat stress also affects cognitive function (i.e., decision-making, attention span, memory, and overall performance) (Schmit et al., 2017), the question remains if cognitive function and performance should be assessed as part of the HTT. There is also the paradigm of possible cognitive heat intolerance that could lead to alterations in body interoception and wrong behavioural responses to a rise in body core temperature. The idea of integrating cognitive testing to the physiological assessment is appealing as it may reflect an interplay between body and mind, but more studies are needed to substantiate it.

**FIGURE 2:**
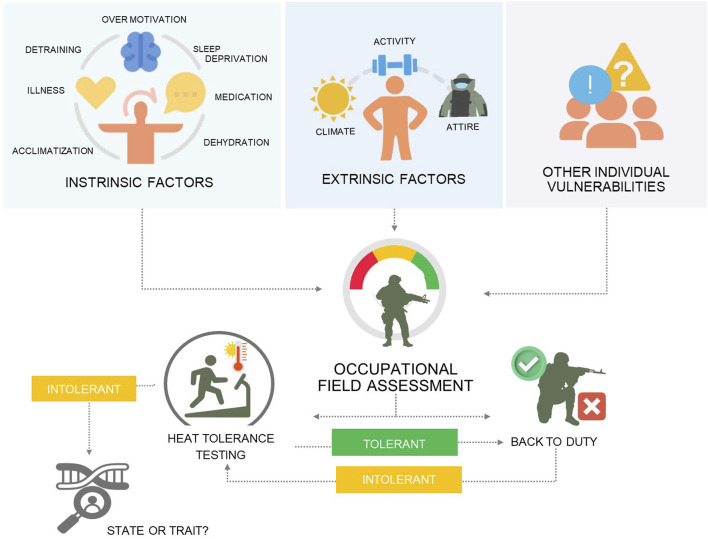
An additive model of incidental risk factors to identify the level of individual vulnerability and the need for heat tolerance before RTD.

An additive model of incidental risk factors to identify individual vulnerabilities, assessment of pre-hospital management and risk factors of the EHS occurrence could be an approach towards deciding the best approach (i.e., function vs. task-oriented testing) and practice to adopt for returning individuals to duty and be assessed in conjunction with potential medical explorations of the origins and reversibility of heat intolerance (Figure 2).

## 7 Conclusion

The significant impact of EHI on people working and training in hot environments is an ongoing global challenge. Acknowledging and maintaining the delicate balance between safety and maximizing performance coupled with growing concerns of increased EHI risk due to rising temperatures is of utmost importance. This has led and will continue to lead the development and implementation of a comprehensive suite of heat management strategies, proactive measures and policies that safeguard the health and performance of men and women of all the various populations that need to train and operate in the heat. The derived evidence-based knowledge and practices from decades of military research can now be harnessed to protect the greater public in face of a warming world.

## Data Availability

The original contributions presented in the study are included in the article/supplementary material, further inquiries can be directed to the corresponding author.
